# Randomness in Sequence Evolution Increases over Time

**DOI:** 10.1371/journal.pone.0155935

**Published:** 2016-05-25

**Authors:** Guangyu Wang, Shixiang Sun, Zhang Zhang

**Affiliations:** 1 CAS Key Laboratory of Genome Sciences and Information, Beijing Institute of Genomics (BIG), Chinese Academy of Sciences, Beijing 100101, China; 2 BIG Data Center, Beijing Institute of Genomics (BIG), Chinese Academy of Sciences, Beijing 100101, China; 3 University of Chinese Academy of Sciences, Beijing 100049, China; Huazhong University of Science and Technology, CHINA

## Abstract

The second law of thermodynamics states that entropy, as a measure of randomness in a system, increases over time. Although studies have investigated biological sequence randomness from different aspects, it remains unknown whether sequence randomness changes over time and whether this change consists with the second law of thermodynamics. To capture the dynamics of randomness in molecular sequence evolution, here we detect sequence randomness based on a collection of eight statistical random tests and investigate the randomness variation of coding sequences with an application to *Escherichia coli*. Given that core/essential genes are more ancient than specific/non-essential genes, our results clearly show that core/essential genes are more random than specific/non-essential genes and accordingly indicate that sequence randomness indeed increases over time, consistent well with the second law of thermodynamics. We further find that an increase in sequence randomness leads to increasing randomness of GC content and longer sequence length. Taken together, our study presents an important finding, for the first time, that sequence randomness increases over time, which may provide profound insights for unveiling the underlying mechanisms of molecular sequence evolution.

## Introduction

The second law of thermodynamics states that a system tends to progress in the direction of increasing entropy [1], where a system in this context includes engineered devices as well as biological organisms and entropy is a measure of randomness; that is to say, a system naturally progresses from nonrandomness to randomness [2]. Consistently, evidence has accumulated that the diversity and complexity in biology tend to increase in any evolutionary system, agreeing well with the second law of thermodynamics [3–7] that randomness never decreases over time. At the molecular level, genome sequences during evolution evolve toward incorporating more intricate mechanisms, indicative of increasing entropy and complexity. Additionally, aging is at least partially due to an accumulation of errors in DNA [8], which can be also explained by an increase in randomness. Considering that cancer can be considered as an evolutionary process [9, 10], mutations and epigenetic imbalances during cancer progression can lead to randomness increase [11, 12], which also consists with the second law of thermodynamics. Therefore, characterizing the dynamics of molecular sequence randomness is of great significance for providing profound insights in unveiling the underlying mechanisms in molecular sequence evolution.

Over the past several years, efforts have been devoted to detecting randomness on molecular sequences primarily at the protein level [13–20]. However, it remains unknown whether DNA sequence randomness changes over time and whether this change consists with the second law of thermodynamics. Specifically, previous studies converted amino acid sequences into bit sequences, based on different groupings of amino acids according to their physicochemical properties, such as size, hydrophobicity, charge, polarity, mass, etc. However, they adopted different physicochemical properties for conversion of amino acid sequences into bit sequences, thus lacking a widely accepted conversion that can be used for randomness detection. In addition, previous studies ignored the degeneracy of the genetic code, that is, amino acids are encoded by different *n*-fold degenerate codons that often have completely different features. For example, CGN (N = A, T, G, C) and AGR (R = A, G) encode Arg, but the former presents higher GC content than the latter.

Based on our previous studies [21–25], codons are not randomly allocated in the genetic code, which can be divided into two halves in a more straightforward and informative manner (Table 1), viz., pro-robustness half (PRH) and pro-diversity half (PDH) that represent robustness and diversity, respectively. Specially, codons in PRH are robust to nucleotide changes at the 3rd codon position (cp3) since they do not provoke the amino acid change (e.g., CCN codes for Pro, where N represents any nucleotide). Conversely, codons in PDH are sensitive to nucleotide changes at cp3; nearly most changes between purines and pyrimidines at cp3 lead to amino acid change (e.g., GAR codes for Glu and GAY codes for Asp, where R = purines and Y = pyrimidines). Although there are three amino acids (Arg, Leu and Ser) encoded by six-fold degenerate codons, they are distributed across the two halves, playing important balancing roles for error minimization [25]. Considering that robustness and diversity are two important features, therefore, it would be desirable to detect sequence randomness based on PDH and PRH and investigate whether a sequence is able to keep a balance between robustness and diversity. As molecular sequences accumulate mutations during evolutionary process, will sequences change the degree of randomness over time? Is this change consistent with the second law of thermodynamics, that is, sequence randomness increases over time?

**Table 1:** The content-centric re-organization of the genetic code.

		1^st^ base			
		A	T	G	C
	A	AAR(K)	TAR(St)	GAR(E)	CAR(Q)
		AAY(N)	TAY(Y)	GAY(D)	CAY(H)
2^nd^ base	T	ATR(I, M)	TTR(L)	**GTN(V)**	**CTN(L)**
		ATY(I)	TTY(F)		
	G	AGR(R)	TGR(St, W)	**GGN(G)**	**CGN(R)**
		AGY(S)	TGY(C)		
	C	**ACN(T)**	**TCN(S)**	**GCN(A)**	**CCN(P)**

Note: N represents any nucleotide. R represents A and G. Y represents T and C. St indicates stop codon.

To address these issues, here we investigate molecular sequence randomness based on a collection of eight statistical random tests. The availability of multiple strains’ genome sequences for a given species provides opportunity to systematically track sequence randomness over time as genes presenting in all related strains are believed to be evolutionarily ancient and those presenting in individual strains are relatively young [26, 27]. Therefore, we collect a total of 61 *Escherichia coli* strains and explore the sequence randomness in the context of pan-genome where genes are classified into different groups according to their presence in different number of strains. As essential genes are more evolutionarily conservative and ancient than non-essential genes [27], we also perform similar analysis by grouping genes based on gene essentiality. We further investigate GC content and sequence length that are in close association with sequence randomness.

## Methods

### Conversion of coding sequences into bit sequences

Following by previous studies [14, 19, 20], biological sequences are converted into bit sequences, which is of practical significance for making randomness detection doable that can rely on many empirical statistical tests (such as The Runs Test, The Random Walker Test and The Serial Test). According to our previous studies [21–24], the genetic code can be re-organized based on both GC and purine contents and accordingly divided into two halves (Table 1), viz., PRH and PDH. Based on these two halves, coding sequences can be converted into bit sequences, where ‘0’ represents a codon in PRH and ‘1’ represents a codon in PDH.

### Randomness testing of bit sequences

A bit sequence is composed of a series of ‘0’ and ‘1’ [28]. Various statistical tests have been proposed to test a null hypothesis that biological bit sequences are random [13, 14, 16, 17, 20, 28–30]. Among them, the National Institute of Standards and Technology (NIST) 800–22 Statistical Test Suite is widely used for random sequence testing. The NIST Statistical Test Suite includes sixteen tests to assess the randomness of binary sequences and each test focuses on a particular characteristic of binary random sequence (S1 Table). Since some tests require sequences longer than 10^5^ (which cannot be always satisfied for sequences in prokaryotes) and thus are inapplicable in biological sequences, we adopt a total of 8 statistical tests (viz., the Frequency Test, the Cumulative Sums Test, the Cumulative Sums Test Reverse, the Runs Test, the Discrete Fourier Transform Test, The Non-overlapping Template Matching Test, The Serial Test, The Approximate Entropy Test; see details in S1 Table), to examine the randomness of coding sequences.

As there are 8 statistical tests used for randomness detection, an 8-dimension vector is employed to describe a sequence, where each dimension represents a *P*-value that is derived from a randomness test. For any given coding sequence *X*, its general randomness vector *R*_*x*_ is formulated as

$$R_{x}=(S_{x}^{1},S_{x}^{2},\cdots ,S_{x}^{8}),$$ (1)

where $S_{x}^{i}$ is the rounded value of negative e natural logarithm of *P*-value in the *i*^th^ random test.

Since any sequence can be represented as an 8-dimension randomness vector, we developed a two-step clustering algorithm [30] based on randomness vectors to cluster sequences into different groups. The first step is to measure the similarity of different sequences using log-likelihood distances and then to cluster sequences into multiple groups with a maximized log-likelihood function. The second step is to further cluster groups by a standard agglomerative clustering method, i.e., comparing their distances to a threshold, and then to determine the best number of clusters based on Schwarz's Bayesian Inference Criterion (BIC) [31].

### Data collection

All coding sequences of 61 *E*. *coli* strains were downloaded from NCBI (National Center for Biotechnology Information) [32]. Essential genes of *E*. *coli* were retrieved from DEG (Database of Essential Genes; http://www.essentialgene.org) [33]. To avoid stochastic errors, sequences that are less than 100bp were removed from analysis. Detailed information can be found at S2 Table.

## Results and Discussion

### Detection of randomness in molecular sequences

**Fig 1:**
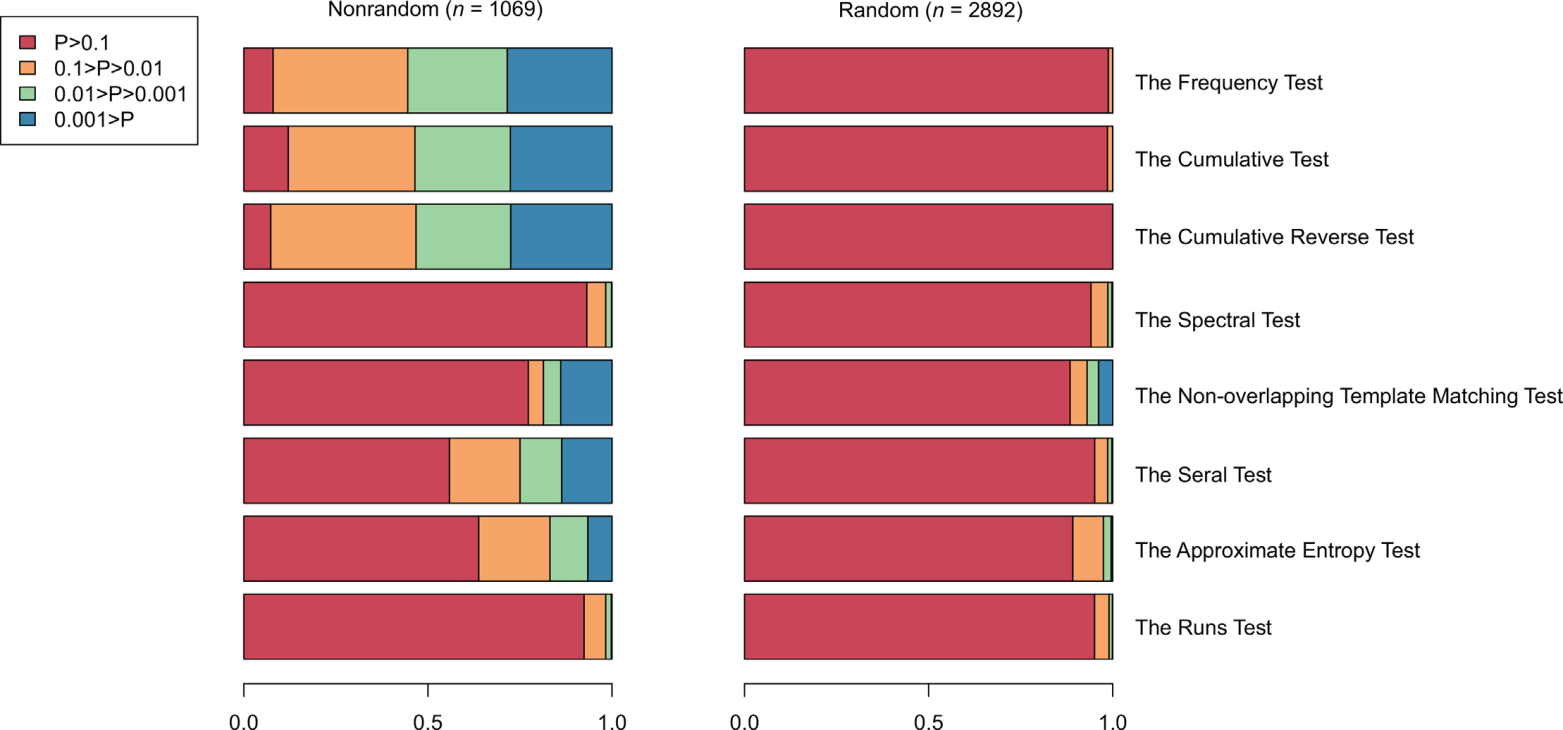
Random and non-random clusters based on 8 statistical tests. Bars are color-coded by different ranges of *P*-value.

To fully capture sequence randomness, we integrate a collection of 8 statistical tests to detect randomness in molecular sequences according to a content-centric organization of the genetic code that splits codons into PDH and PRH (Table 1; see Methods). Based on these 8 tests, we devise an 8-demension vector, where each dimension represents a *P*-value derived from a randomness test. As a result, any sequence can be denoted as an 8-dimension randomness vector. We further develop a two-way clustering algorithm based on randomness vector and apply it to all sequences in *E*. *coli* MG1655, leading to two clusters with distinct statistical properties of randomness (Fig 1): the random cluster (*n* = 2,892) and the nonrandom cluster (*n* = 1,069). Detailed information of statistical testing on these two clusters is tabulated into S1 and S2 Tables. Considering the significance levels of 8 statistical tests, the random cluster has a higher percentage (>89.42%) of sequences whose statistical significance levels are larger than 0.1, clearly showing that the majority of sequences in this cluster have random patterns. Contrastingly, the nonrandom cluster contains a larger proportion of sequences that have significance levels less than 0.1 (Fig 1). Intriguingly, the runs test performs very similar in both clusters. This result is in agreement with a previous finding that the runs test is unable to detect randomness in biological sequences [18]. Likewise, the spectral test yields similar performances in both clusters, indicating its incapability in detecting randomness biological sequences as well.

### Investigation of sequence randomness over time

A pan-genome represents the union of all gene sets in all available strains of a species, which includes core genes that are present in all strains and dispensable genes that are present in multiple but not all strains [34]. As core genes are believed to be more ancient [26], therefore, we hypothesize that sequence randomness increases over time and core genes most likely contain more randomness.

**Fig 2:**
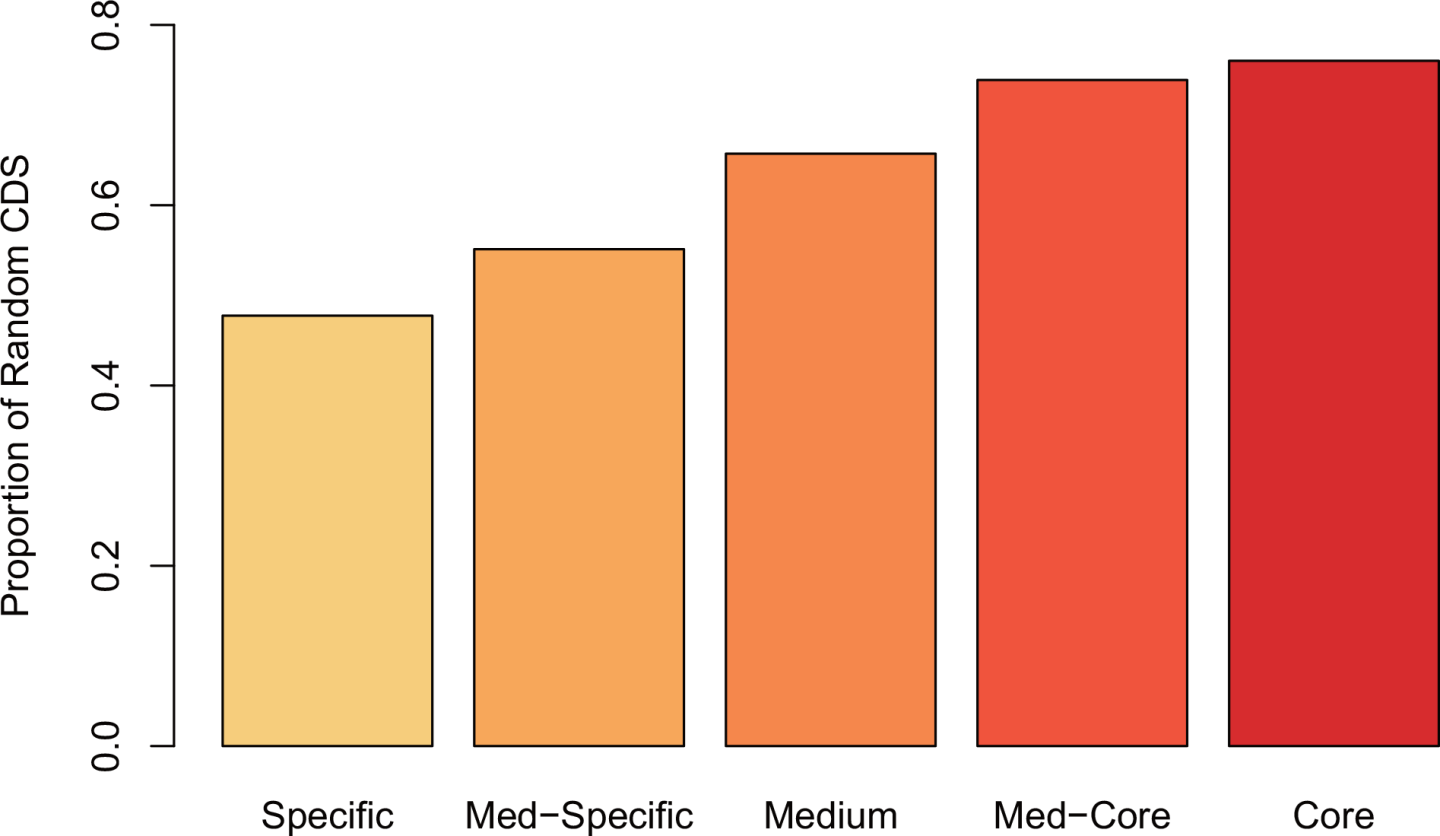
Proportion of random sequences in *E*. *coli*.

To test this hypothesis, we collect 61 publically available *E*. *coli* genomes from [35] (S2 Table), perform the pangenome analysis and classify genes of *E*. *coli* MG1655 into five groups according to their presence in these 61 strains: Specific (that are genes presenting in 1–15 strains; *n* = 111), Medium-Specific (that are genes presenting in 16–30 strains; *n* = 126), Medium (that are genes presenting in 31–45 strains; *n* = 315), Medium-Core (that are genes presenting in 46–60 strains; *n* = 1,347) and Core (are genes presenting in all 61 strains; *n* = 2,060). Consistent with our expectations, the proportion of random genes is significantly different in these five groups (Chi-square test, *P*<0.0001; Fig 2) and grows gradually from specific genes to core genes, exhibiting 47.75% in specific genes and reaching the highest at 76.02% in core genes. As core genes are more ancient whereas specific genes are relatively young [26], these results clearly show that sequence randomness increases over time.

To further validate our results, we perform similar analysis by considering gene essentiality since essential genes that are critical for an organism’s survival are thought to be more ancient [26, 36, 37]. We retrieve 527 essential genes and 2,956 non-essential genes from DEG (Database of Essential Genes) [38]. In contrast to core genes that are derived from computational analysis, essential genes derived from DEG are identified by experimental approach. Consistently, a chi-square test of independence demonstrates that essential genes have a significant excess of random genes compared with non-essential genes (*P*<0.0001; Table 2). Ribosome proteins play a significant role in translation machinery and are believed to be more ancient than others [39]. We find that the majority of ribosome proteins (74%; S3 Table) are random, consisting well with our results that old genes are more random. Taken together, these results collectively demonstrate that randomness in molecular sequence increases over time. As randomness is detected based on grouping codons into PRH and PDH, an increase in sequence randomness during evolution leads to a uniform usage of codons in these two halves (Table 3), suggesting that sequences evolve toward achieving a good balance between robustness and diversity.

**Table 2:** Statistical test of randomness between essential and non-essential genes.

Cluster	Essential Genes	Non-essential Genes	2×2*χ*^2^*P*-value
Random	418	2120	<0.0001
Nonrandom	109	836	

**Table 3:** Percentage of genes that equally use codons in PDH and PRH.

Pan-genome group	Percentage*
Core	77.6%
Medium-Core	75.9%
Medium	65.4%
Medium-Specific	53.5%
Specific	46.8%

* *P*-value<0.05 (The frequency test)

### Variation of GC content and sequence length over time

**Fig 3:**
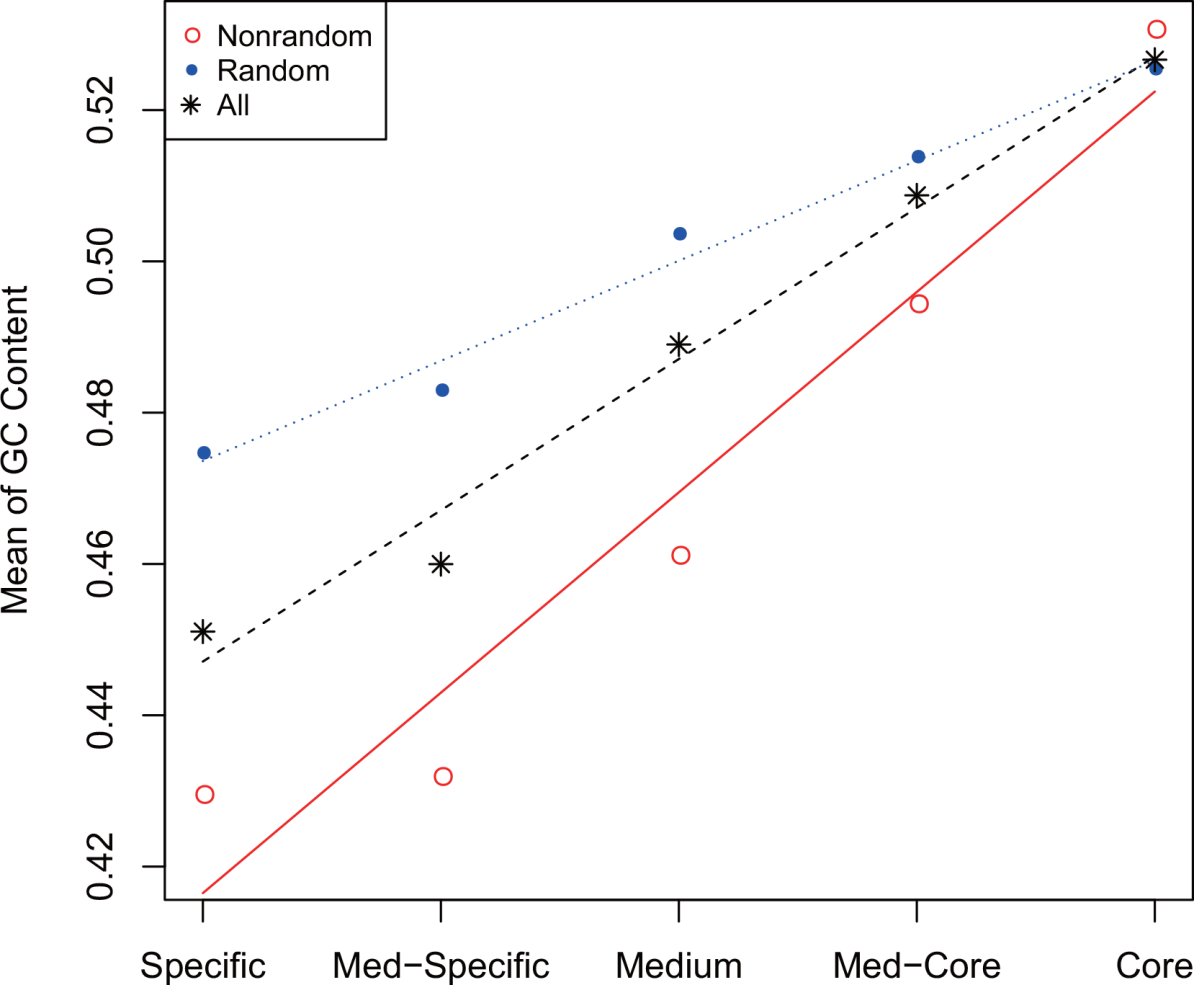
Variation of GC contents in the *E*. *coli* pan-genome. Random and nonrandom sequences are examined separately and each dot represents the average of GC content across a specific gene set.

As sequence randomness increase may provoke random nucleotide composition, we further test whether GC content becomes more random over time. If nucleotide composition in one gene is random, its GC content is expected to be around 0.514 (≈ (96–2) / (64×3–3×3) after removal of three stop codons). Therefore, we compare GC contents of random and nonrandom sequences and investigate their variations in the pan-genome context (Fig 3). Our results show that random sequences present GC contents significantly different from nonrandom sequences (t-test, *P*<10^−14^; Fig 3); GC content in random sequences fluctuates around 0.51, always higher than that in nonrandom sequences, and intriguingly, such pattern is strikingly apparent in specific genes. This result is consistent well with a previous study that GC content in old human genes is around 0.51 [40]. With the increasing presence in more *E*. *coli* strains, the difference of GC content between random and nonrandom genes is radically reduced. These results show that GC content indeed goes random over time; GC content in random sequences varies within a very narrow range around 0.51, strongly indicating that random sequences achieve robustness-diversity balance.

**Fig 4:**
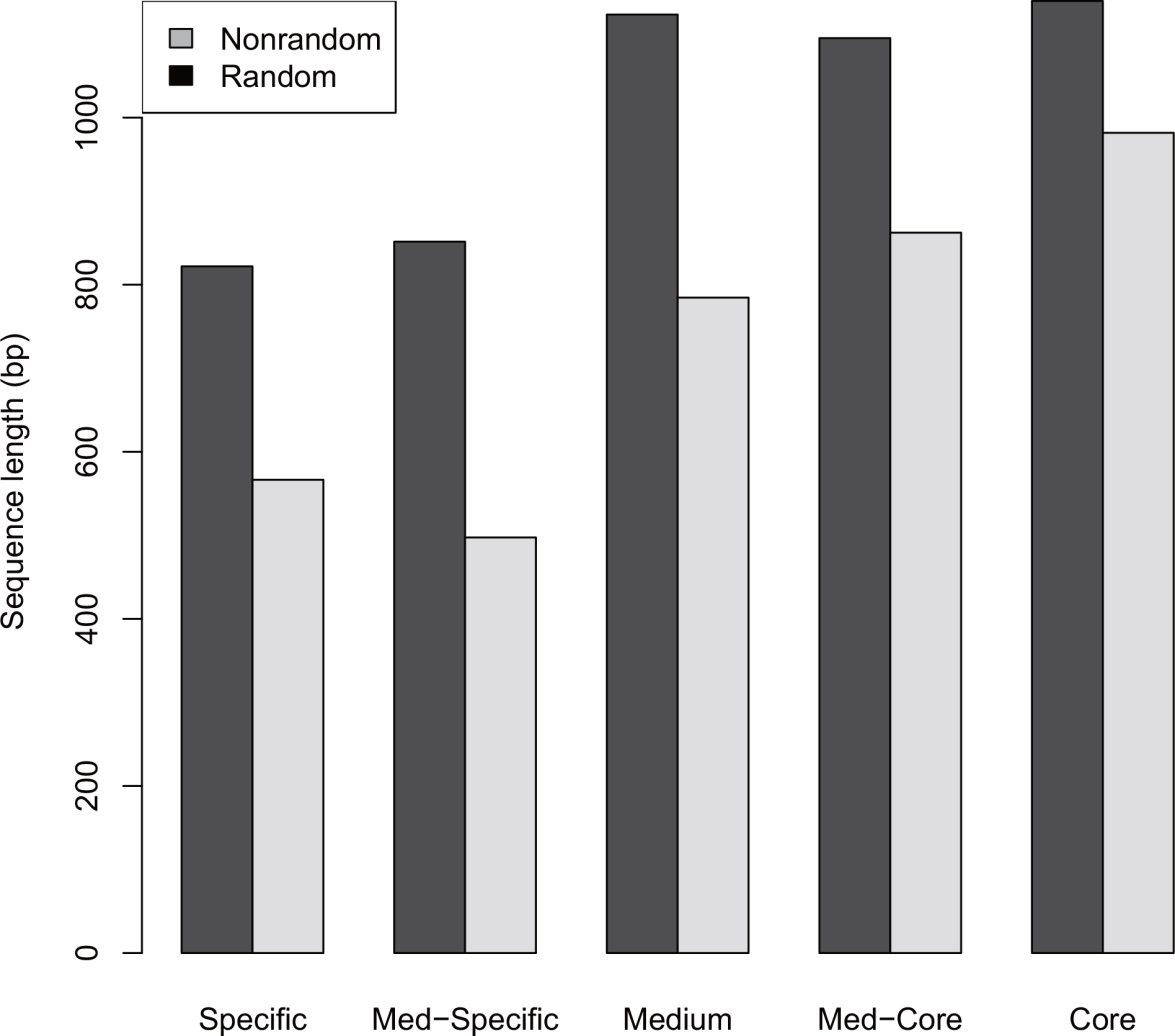
Length of coding sequences in the *E*. *coli* pan-genome. Random and nonrandom sequences are examined separately and each bar represents the average of sequence length across a specific gene set.

It has been extensively reported that GC content is correlated positively with sequence length [41–43]. Therefore, we wonder whether sequence length varies over time (Fig 4). Agreeing with expectations, core genes are longer than specific genes and therefore, sequence length increases over time. In addition, random genes tend to be always longer than nonrandom genes. Collectively, with the increase of sequence randomness during evolution, sequences evolve toward higher GC content fluctuating at random and possess longer length, which is more pronounced in random sequences.

## Conclusion

To fully picture the dynamics of randomness in molecular sequence evolution, here we detected sequence randomness in *E*. *coli* and explored randomness variation over evolutionary time based on the fact that in the context of pan-genome core genes are more ancient. Consistent with the second law of thermodynamics, we found that core genes are more random than specific genes, indicating that randomness in molecular sequence increases over time. Moreover, this conclusion still holds true when we considered gene essentiality, given that essential genes are more conservative and ancient than non-essential genes. To our knowledge, our study presents an important finding, for the first time, that randomness in sequence evolution increases over time, coupled with an increase in randomness of GC content and longer sequence length, which needs further validation in a wide range of species across three domains of life.

## Supporting Information

S1 Table Characteristics of the NIST Statistical Tests. (XLS)

S2 Table 61 publically available *E*. *coli* genomes. (XLS)

S3 Table Ribosomal proteins in Pan-genome group and Random group. (XLS)

S4 Table Proportion of Each Test in Random Group. (XLS)

S5 Table Proportion of Each Test in Nonrandom Group. (XLS)
