# Correction: Randomness in Sequence Evolution Increases over Time

**DOI:** 10.1371/journal.pone.0158191

**Published:** 2016-06-20

**Authors:** Guangyu Wang, Shixiang Sun, Zhang Zhang

There are errors in the Funding section. The correct funding information is as follows: This work was supported by grants from National Programs for High Technology Research and Development [863 Program; 2014AA021503, 2015AA020108] and The 100-Talent Program of Chinese Academy of Sciences. The funders had no role in study design, data collection and analysis, decision to publish, or preparation of the manuscript.
